# Pouring petrol on the flames: Using oncolytic virotherapies to enhance tumour immunogenicity

**DOI:** 10.1111/imm.13323

**Published:** 2021-03-28

**Authors:** Alicia Teijeira Crespo, Stephanie Burnell, Lorenzo Capitani, Rebecca Bayliss, Elise Moses, Georgina H. Mason, James A. Davies, Andrew J. Godkin, Awen M. Gallimore, Alan L. Parker

**Affiliations:** ^1^ Division of Cancer and Genetics Cardiff University School of Medicine Cardiff University Cardiff UK; ^2^ Division of Infection and Immunity Cardiff University School of Medicine Cardiff University Cardiff UK

**Keywords:** antibodies, CAR T cells, immunotherapy, model systems, oncolytic viruses, virotherapy

## Abstract

Oncolytic viruses possess the ability to infect, replicate and lyse malignantly transformed tumour cells. This oncolytic activity amplifies the therapeutic advantage and induces a form of immunogenic cell death, characterized by increased CD8^ +^ T‐cell infiltration into the tumour microenvironment. This important feature of oncolytic viruses can result in the warming up of immunologically ‘cold’ tumour types, presenting the enticing possibility that oncolytic virus treatment combined with immunotherapies may enhance efficacy. In this review, we assess some of the most promising candidates that might be used for oncolytic virotherapy: immunotherapy combinations. We assess their potential as separate agents or as agents combined into a single therapy, where the immunotherapy is encoded within the genome of the oncolytic virus. The development of such advanced agents will require increasingly sophisticated model systems for their preclinical assessment and evaluation. In vivo rodent model systems are fraught with limitations in this regard. Oncolytic viruses replicate selectively within human cells and therefore require human xenografts in immune‐deficient mice for their evaluation. However, the use of immune‐deficient rodent models hinders the ability to study immune responses against any immunomodulatory transgenes engineered within the viral genome and expressed within the tumour microenvironment. There has therefore been a shift towards the use of more sophisticated ex vivo patient‐derived model systems based on organoids and explant co‐cultures with immune cells, which may be more predictive of efficacy than contrived and artificial animal models. We review the best of those model systems here.

## Graphical abstract

Oncolytic virotherapies have immense potential to ‘heat up’ otherwise immunologically ‘cold’ tumour types. In this review, the capacity of virotherapies to synergize with or express a range of types of immunotherapies is explored. Optimizing such combinations in preclinical models is complex, and we therefore review the most clinically relevant rodent and ex vivo human models available for such assessment.

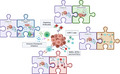

**Abbreviations**
BiKE
: Bispecific NK cell engagers
BiTE: Bispecific T ‐cell engaging
CAF
: cancer‐associated fibroblast
CAR: chimeric antigen receptor
cBITE
: EGFR‐targeting BITE
CEA: carcinoembryonic antigen
CTLA4
: cytotoxic T‐lymphocyte‐associated protein 4
DNA: deoxyribonucleic acid
EGFR
: epidermal growth factor receptor
EMA: European Medicines Agency
EnAd
: enadenotucirev
EpCAM: epithelial cell adhesion molecule
FAP: fibroblast ‐activating protein
FDA
: Food and Drug Administration
GM‐CSF: granulocyte‐macrophage colony‐stimulating factor
GSC: glioblastoma stem cell
HER2: human epidermal growth factor receptor 2
HSCs: haematopoietic stem cells
ICI
: immune checkpoint inhibitor
IL‐2: interleukin‐2
IL‐12
: interleukin‐12
IL‐15: interleukin‐15
ImmTACs
: immune mobilizing monoclonal T‐cell receptors against cancer
mAb: monoclonal antibody
MHC: major histocompatibility complex
NDV
: Newcastle disease virus
NK: natural killer
OV: oncolytic virus
PD‐1: programmed cell death protein 1
PDE
: patient‐derived explant
PDX: patient ‐derived xenograft
pHLA
: human leucocyte antigen peptide
RNA: ribonucleic acid
ScFv: single ‐chain variable fragment
TAA
: tumour‐associated antigen
TCR: t‐cell receptor
TME: tumour microenvironment
Treg
: T regulatory
T ‐VEC: talimogene laherparepvec
VDEPT: virus ‐directed enzyme prodrug therapy

## INTRODUCTION

Whilst it is clear the immune system can recognize and kill cancer cells, it is evident that for the most part that cancers have evolved many mechanisms for evading immune attack. Whilst current immunotherapies, such as checkpoint inhibitors and cellular therapies, can overturn or overcome these mechanisms, they are only successful in certain types of cancer and only in a minority of patients. There is, however, tremendous scope for improvement through a better understanding of the barriers to immune attack and development of novel methods for stimulating effective anti‐cancer immune responses. As discussed below, oncolytic viruses are poised to offer answers to both challenges in that they can be engineered to specifically infect cancer cells whilst simultaneously delivering immune ‐enhancing therapies selectively at the site of infection.

## ONCOLYTIC VIRUSES

**FIGURE 1:**
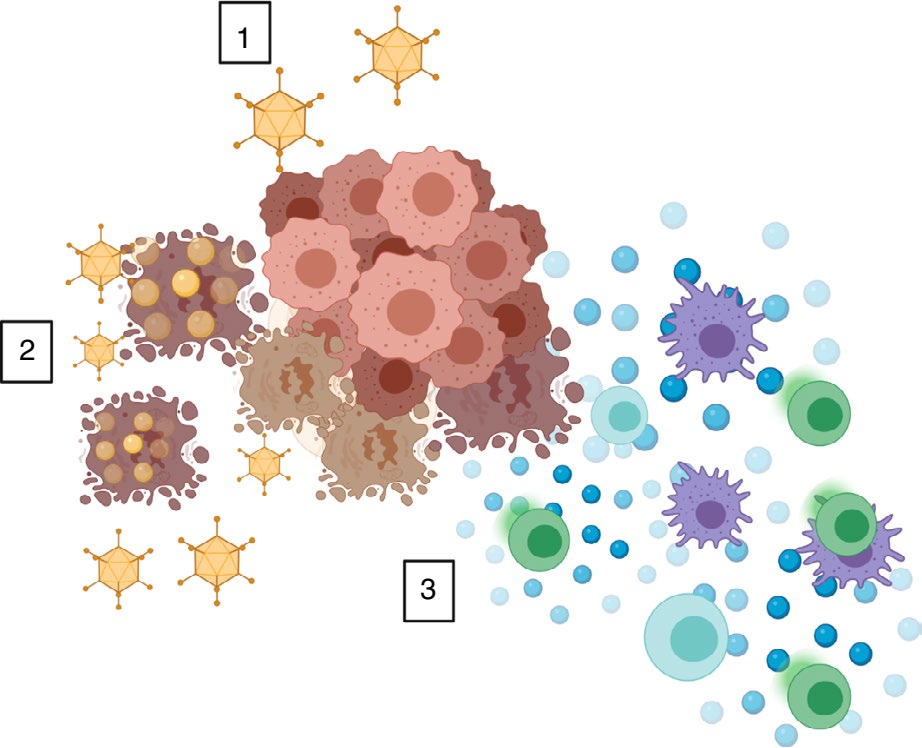
Oncolytic viruses as a cancer therapeutic. Oncolytic viruses (OVs) can be engineered to selectively recognize tumour cells (1), replicate within those infected cells to produce thousands of daughter virions (2) and lyse tumour cells, releasing tumour antigens into the tumours microenvironment, where they can be processed by dendritic cells and presented to T cells (3)

The use of oncolytic viruses (OVs) as anti‐cancer therapeutics offers potential to break tumour tolerance. Although some viruses have naturally improved ability to replicate within cancer cells, most OVs are engineered agents that have been refined to selectively infect or replicate within transformed cells. A wide range of OVs are under development, with those based on adenovirus, herpes simplex virus, reovirus, vaccinia virus, measles virus, Coxsackie virus and Newcastle disease virus (NDV) proving effective at the preclinical level, with some progressing to clinical trials [1, 2, 3]. Unfortunately, whilst efficacy as a monotherapy has been disappointing, development as combination therapies has yielded more promising outcomes especially in combination with immunotherapies. Typically, viruses are small, infectious agents containing either DNA or RNA genomes. In their wild‐type state, they are often pathogenic, although, through refinement of the genome, they can be manipulated to replicate within malignantly transformed cells and also to bind selectively to receptors overexpressed in cancer cells [4], enabling selectivity at the level of cellular infection (Figure 1). Tightly controlled tumour selectivity is a key consideration, since optimally refined OVs will result in minimal uptake in ‘off‐target’ tissues. Uptake by non ‐transformed healthy cells depletes the pool of OV to ‘off‐target ’ tissues, limiting the bioavailability of OV for active tumour targeting. These major challenges in the OV field in achieving tumour‐selective systemic delivery of OVs have seen significant progress in recent years with the development of ‘ precision virotherapies’, although significant challenges remain [5, 6]. These advances and current challenges have been recently and extensively reviewed elsewhere [7, 8, 9].

An additional appealing feature of oncolytic viruses is the capacity of the viral genome to encode therapeutic transgenes. Early studies focussed on transgenes that were indirectly toxic to tumour cells, in particular the use of ‘virus‐directed enzyme prodrug therapy’ (VDEPT). A notable example of this is nitroreductase [10], which converts the nitrogen mustard prodrug CB1954 into a DNA cross‐linking agent. Despite the safety and tolerability of this approach, efficacy is limited for a variety of reasons including low transfection/transduction efficiency of the vectors, non‐specific toxicity and slow prodrug–drug conversion rate [11]. Another promising avenue has involved incorporation of transgenes encoding cytokines such as IL‐12, IL‐2, IL‐15 and GM‐CSF within the OV genome to stimulate the recruitment of immune cells to the tumour microenvironment (TME). These OVs have demonstrated significant potential to treat various cancers [12 –15], and evidence of their potential is suggested in the fact that both the FDA and the EMA have already licensed talimogene laherparepvec (T‐VEC, Imlygic™), a modified herpes simplex virus (HSV) expressing GM‐CSF, for the localized treatment of malignant melanoma [16 ]. A significant limitation of HSV‐based OVs is that their efficacy appears to be limited to local intratumoral administration, which limits practical clinical application to those approaches where local delivery of therapeutic is feasible. An ideal OV would be highly targeted to malignantly transformed cells following intravascular administration, and able to efficiently localize to and infect metastases in patients with advanced forms of disease.

The immunogenic nature of cell death induced by an OV has significant promise in sensitizing tumours to immunotherapies [4, 12, 17]. Building on the improved understanding of the role of the immune system in the control of tumour growth, OVs have been used either in combination with immunotherapies or armed with immunological transgenes to stimulate the host anti‐tumour immune responses. In this review, we outline some of the most promising forms of immunotherapies that might form part of the increasingly sophisticated ‘ immunovirotherapy’ repertoire moving forward, and the potential model systems that might be best employed to evaluate them.

## DELIVERING IMMUNOTHERAPIES USING ONCOLYTIC VIRUSES

**FIGURE 2:**
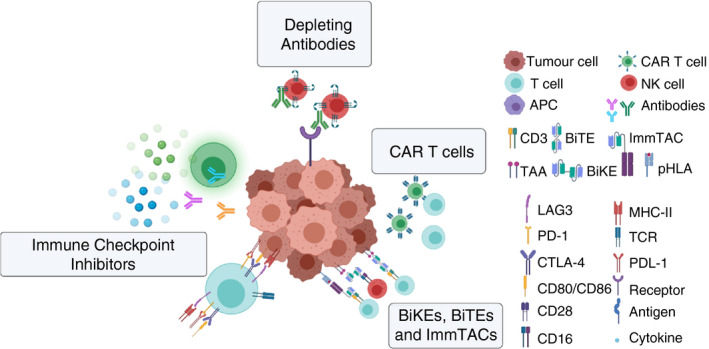
Current immunotherapies selectively enhance immune response. Immune checkpoint inhibitors bind to and inactive negative regulators of immunity such as CTLA‐4 and PD‐L1. Depleting antibodies recognize tumour antigens and can be administered directly to a patient, where they bind the TAA on tumour cells and stimulate immune cell activation. CAR T cells are generated by removing T cells from cancer patients, genetically transforming them using a viral vector to present chimeric antigen receptors targeted selectively to TAAs, expanded ex vivo and reinfused into the patient. Bispecific molecules, BiKEs, BiTEs and ImmTACs, target the interaction between the T cell or NK cell and the TAA/pHLA presented on the tumour cell and physically synapse the two, activating the T cell and resulting in immune‐ mediated tumour cell killing

Until recently, the mainstay cancer treatments were limited to combinations of chemo‐radiotherapy, surgery and targeted therapies. Although advances in each of these treatments have sought to minimize side‐effects, these remain a significant issue [18]. It has become clear that immunotherapy, most notably immune checkpoint inhibitors (ICIs), chimeric antigen receptor (CAR) T cells, depleting monoclonal antibody (mAb) therapies and bispecific molecules, is no exception, with many patients experiencing severe side‐effects characterized by the onset of autoinflammatory and autoimmune diseases [19, 20, 21], arising as a result of non‐specific immune stimulation and off‐target effects. There is therefore great potential for using OVs to improve the safety and specificity of these treatments, mainly by allowing the therapeutic to be delivered directly and specifically to the tumour (Figure 2 ).

## THERAPEUTIC ANTIBODIES

Immune checkpoints, most notably cytotoxic T‐lymphocyte‐associated protein 4 (CTLA‐4) and programmed cell death protein 1 (PD‐1), comprise an important part of homeostatic pathways crucial for the maintenance of peripheral tolerance and the regulation of immune responses [22]. ICIs block these homeostatic signals and attempt to induce new immune responses or ‘re‐invigorate’ the ‘exhausted’ immune response towards tumours [22] (Figure 2). Although the potential of ICIs is established, particularly in melanoma patients receiving a combination of PD‐1 and CTLA ‐4 blockade, the percentage of people who can benefit from this type of therapy remains low [23, 24]. In this context, virotherapies may provide significant immune‐enhancing effects. Indeed, combination of immunotherapies with virotherapy has demonstrated promise in treating cancers by overcoming tumour resistance to ICIs allowing effective anti‐tumour responses to develop [25, 26, 27, 28, 29]. Chon et al.[29] demonstrated that the OV mJX‐594 was able to sensitize ICI‐resistant tumours and promote significant T‐ cell infiltration into tumours in mice, and, in combination with anti‐PD‐ 1 therapy, reduced tumour growth by a 70%. Similarly, Zamarin et al.[27] demonstrated that protection against tumour rechallenge doubles when treated with NDV and anti‐CTLA‐4 combination therapy compared with mice treated with anti‐CTLA‐4 therapy alone, enhancing tumour lymphocyte infiltration. Encouragingly, similar outcomes have also been demonstrated in human trials. During the clinical trial to treat stage IIB‐IV melanoma, Puzanov et al.[30] studied the immune response in patients treated with T‐VEC and ipilimumab, observing limited therapeutic responses in monotherapy trials, whilst the combination demonstrated increased CD4^+^ICOS^+^ T cells were associated with significantly improved therapeutic outcomes. At the time of writing, a phase 3 clinical trial studying the combination of pembrolizumab (anti‐PD‐1) with and without T‐Vec has just completed, the results of which are eagerly anticipated (NCT02263508). These studies demonstrate the significant potential to combine the self ‐amplifying ability of virotherapies with the local tumour selectivity of immunotherapies to enhance anti‐tumour immune responses. The potential synergy of OVs with ICIs has made their combination use in clinical trials popular, and a wide range of combinations are currently being assessed [31]. An extensive overview of these combination trials is provided in Table S1.

Oncolytic virus represents excellent candidates to increase the amount of antibody produced locally at the site of the tumour. Resistance to antibody therapies can be acquired as a result of modifications to the cellular phenotype [32] and accelerated by exposure to subtherapeutic levels of the antibody [33, 34]. This is facilitated by physical characteristics of the TME, such as the presence of a high hydrostatic pressure that reduces the penetration of antibodies from the systemic circulation [35], internalization and endocytic clearance occurring at the edges of tumours [ 36]. Such factors can result in poor distribution, with various studies highlighting the need to improve the penetrance to improve treatment efficacy [37]. Due to their tumour selectivity, OVs encoding antibodies could aid in circumventing these hurdles by inducing the production of therapeutic antibodies locally within tumours themselves. There are over 50 mAb therapies approved to date, which could be explored. These antibodies include the well‐publicized checkpoint inhibitors such as anti ‐CTLA‐4, which may also derive some therapeutic effect from the depletion of Tregs within the tumour environment [38, 39, 40]. To date, only a limited number of OV expressing ICIs have undergone clinical evaluation (overviewed in Table 1), but the number entering trials are certain to increase rapidly as technologies improve to ensure tightly regulated tumour selectivity overexpression of ICIs.

**TABLE 1:** Overview of clinical trials utilizing oncolytic viruses expressing immune checkpoint inhibitors as transgenes

OV Type	Transgene expressed	Tumour type	Clinical phase	Trial Ref
Adenovirus	Biological: CAdVEC (PD‐1 minibody)	Solid tumours	Phase 1	NCT03740256
Herpes simplex virus	Biological: NG34scFvPD‐1 (scFvPD ‐1)	Glioblastoma	–	C. Passaro et al.[78]
	Biological: RP2 (CTLA‐4 antibody)		Phase 1	NCT04336241

## CAR T CELLS

**FIGURE 3:**
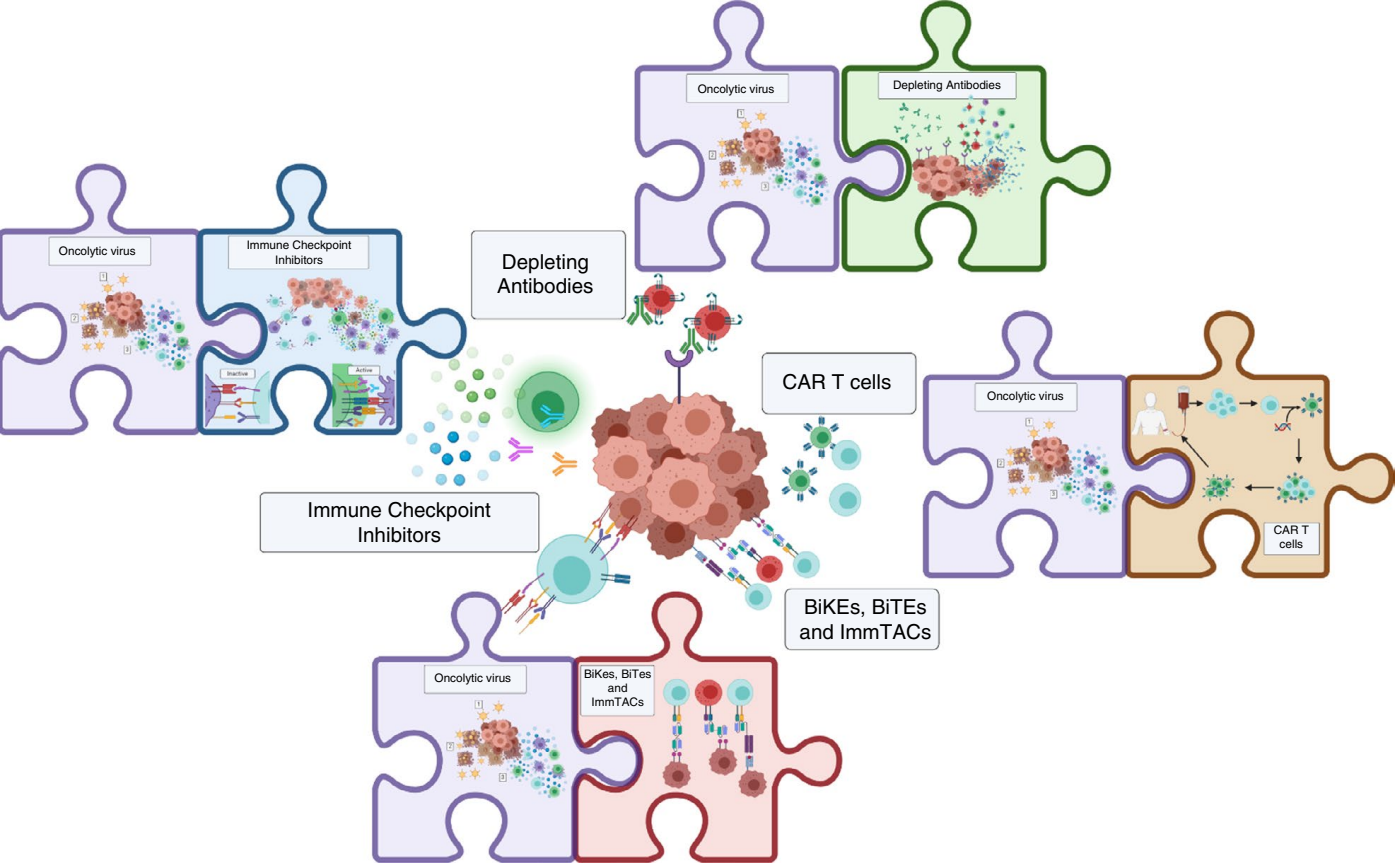
Oncolytic viruses, pieces of the puzzle. OVs can direct immunotherapies such as ICI, depleting antibodies, CAR T cells and bispecific antibodies to the tumour and consequently reduce off‐target and dose‐dependent toxicity, thereby increasing the efficacy of the treatment

Chimeric antigen receptor T cells, comprising genetically engineered T cells that express single‐chain antibodies specific for tumour antigens linked to signalling adaptors of the T‐ cell receptor (TCR) (eg the ζ chain of the CD3 complex) [41, 42] (Figures 2 and 3), have also shown significant successes in the context of haematological malignancies [43]. Treatment of solid tumours, however, has been less successful due to TME‐imposed barriers to CAR T‐cell trafficking and infiltration, as well as the lack of good targets presently identified in solid cancers [ 44, 45, 46 ]. However, recent studies engineering an OV to express a truncated form of CD19 on infected tumour cells ‘marked out’ those cells for subsequent treatment with CAR T‐cell therapies, this increased T‐cell tumour infiltration and improved survival in mouse melanoma and colorectal cancer models [47, 48]. The use of CAR T cells as carriers of OV has also been suggested enabling the deposition of virus into the tumour cells, indicating that this combination relationship has the ability to work both ways [ 49]. Such examples provide additional evidence of the scope to tailor OV to niche applications, sensitizing tumour models not only to antibody‐based ICI therapies, but also to CAR T cells.

## BISPECIFIC MOLECULES

Bispecific T‐cell engaging or NK engaging (BiTE or BiKE) proteins are composed of two single‐chain variable Fv fragments of target antibodies connected by a flexible linker that simultaneously binds to T cells or NK cells via an anti‐CD3 or anti‐CD16 antibody, and tumour cells via an anti‐tumour antigen antibody [50]. By engaging either CD3/CD16 or the target cell antigen, T cells or NK cells can be activated, increasing expression of activation markers and resulting in tumour cell lysis independent of antigen recognition and MHC class I expression, which is often downregulated on tumour cells. Bispecifics have had success in a range of preclinical models with BiTEs designed to target TAAs including EGFR, EPCAM, CEA and HER2/neu with some undergoing clinical evaluation [50,51].

**FIGURE 4:**
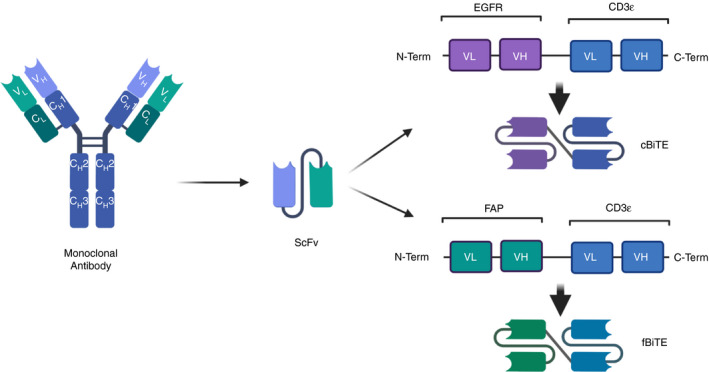
Production of BiTEs expressed by adenovirus. OAd ICO15K is an engineered adenovirus expressing CBiTE (ICOVIR‐15K) or an FBITE (OAd ICO15K ‐FBiTE). BiTEs utilize the ScFv portion of the monoclonal antibody to target different proteins. In this case, the N‐terminal of the BiTE targets either EGFR (cBiTE) or FAP (fBiTE), whilst the c‐terminal of the BiTE was specific for CD3

Although bispecifics have shown promising results, their use may be limited by toxicities, short biological life spans, poor retention at tumour sites and inability to generate a lasting memory immune response [52, 53]. In order to combat this, Fajardo et al. developed an oncolytic adenovirus (ICOVIR‐15K) engineered to express an EGFR‐ targeting BITE (cBITE) (Figure 4). In co‐ culture assays, oncolysis resulted in T‐cell activation, proliferation and cytotoxicity. ICO15K‐cBITE was shown to be tumour‐selective as healthy cells expressing low protein levels had low adenovirus‐mediated cytotoxicity. Intratumoral injection increased persistence and accumulation of tumour‐ infiltrating T cells in vivo compared with parental virus, and combined delivery of ICOVIR‐15K cBiTE with peripheral blood mononuclear cells or T cells enhanced the anti‐tumour efficacy achieved by the parental control in xenograft models [54, 55].

ICOVIR‐15K was further utilized to develop an OAd encoding fibroblast‐activating protein (FAP)‐targeting BiTE (fBiTE). This fBiTE consists of two ScFv, one specific for human CD3ε and the other specific for murine and human FAP assembled with a GS linker (Figure 4) [56]. With this approach, they targeted infiltrated lymphocytes against FAP‐expressing CAFs, simultaneously targeting cancer cells and redirecting immune responses towards the tumour stroma fibroblast to improve tumour permeability and virus spread. A similar approach is the engineered adenovirus enadenotucirev (EnAd), modified to enhance T‐cell activation and recognition of EpCAM‐positive target cells, leading to clustering and activation of both CD4^+^ and CD8^+^ T cells. This promoted endogenous tumour cell killing in primary pleural effusions and peritoneal malignant ascites despite the immunosuppressive TME [57, 58].

To increase the effectiveness of the anti ‐tumour activity of CAR T cells, a combination of OVs and BiTEs has been utilized. CAR T cells targeting folate receptor α can successfully infiltrate pre ‐established xenograft tumours but failed to induce a complete response due to the presence of antigen‐negative tumour cells [59]. As they are antigen‐dependent, generation of an Ad‐BiTE EGFR bispecific that mediated oncolysis significantly improved CAR T‐cell activation and proliferation due to the activation of the CAR T‐cell fraction by the increase in cytokines from the OAd‐BiTE‐infected cells [59].

**FIGURE 5:**
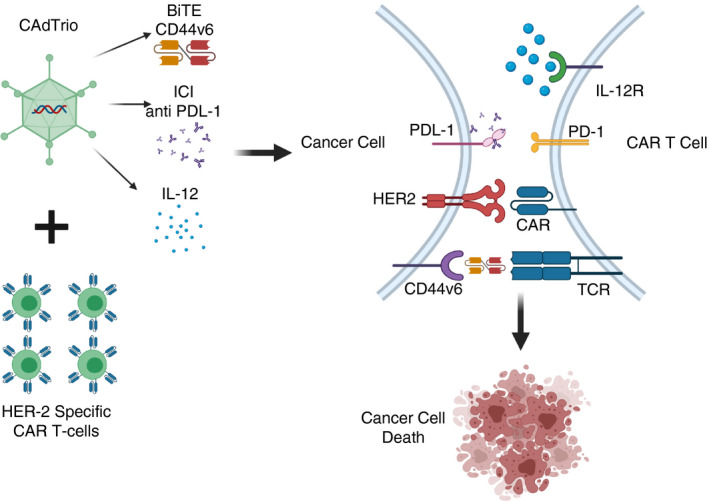
Using viruses to target multiple pathways. The CAdTrio virus is able to produce (i) a BiTE specific for CD44v6^+^ cancer cells, thereby bringing these cells into contact with the T cells via the TCR, (ii) antibodies against anti‐PDL‐1 resulting in the prevention of PD‐ 1/PDL‐1 interaction and in immune checkpoint inhibition and (iii) IL‐12 to stimulate the growth and function of T cells. When this virus is used in combination with HER‐2‐specific T cells, this also induces the interaction between HER2^+^ cancer cells and the CAR molecules on the T cells resulting in the cell death of HER2^+^ and HER2^−/−^ CD44v6 tumours

Oncolytic viruses can be readily engineered to combine different immunotherapies including BiTEs, cytokine production and ICIs. Porter et al.[60] generated a single adenovirus encoding both IL‐12 and anti‐PDL‐1, as well as a BiTE specific for CD44v6. This OV, named CAdTrio, was given to mice with HER‐2‐specific CAR T cells, and this improved tumour control and survival (Figure 5) [60]. Taken together, these findings demonstrate the significant potential for local OV‐ mediated expression of bispecific engager therapies to mediate efficacy across a range of tumour models.

A novel format of bispecific molecules are the immune mobilizing monoclonal TCRs against cancer (ImmTACs) that uses TCR specificity to engage with target cells [61]. Bispecific formats are limited by recognition of cell surface antigens, restricting the repertoire of targets to <10% of all antigens. In comparison, ImmTACs are able to recognize intracellular antigens (>90% of protein‐coding genes) through the TCR via peptide fragments presented by human leucocyte antigen (pHLA) [62]. Unlike BiTEs and CAR T‐cell therapies, ImmTACs are the first bispecific molecule to combine high affinity binding to pHLA with the redirection and activation of non‐tumour‐specific T cells. Whilst current data on ImmTACs combined with OV are limited, it is possible that as with BiTEs and BiKEs, the tumour‐specific expression of ImmTACs from within OV platforms could offer significant advantages around increased potency with reduced toxicity.

## MODEL SYSTEMS

Mechanisms of tumour selectivity are virus‐dependent and need to be determined and proven efficient before these treatments enter the clinic to rule out any adverse effects. At the very least, the model system used to evaluate an OV depends on the OV in question, what is being targeted, the condition being treated, the mechanism of action and whether it is being considered as a mono‐ or combination therapy. Thus, selection of appropriate models for testing and validation will need to take each of these considerations into account.

A major limitation of OVs is the host ‐selective nature of replication, as many of the human‐specific OVs that would be utilized as virotherapies cannot replicate in murine cells and tissues. In order to study off‐target replication toxicity for this virus, only human cells would provide reliable and meaningful results; therefore, a set of preclinical studies using a combination of in vitro safety tests needed to be designed [63]. It is thought that replication and lysis contributes to immunogenicity; however, there is limited evidence that supports significant replication in patients. This will be the case for most OV assessments as combinations of advanced models will be required as discussed below.

Initial validation of OV therapies have been carried out in cell lines to ensure OV is able to specifically replicate in tumour cells or can target certain markers [64]; however, further information regarding the TME and immune response requires the use of more complex systems. The most commonly used test system is the immunocompromised mouse model, either using cell lines or patient‐derived xenografts (PDX) to produce the target tumour. Immunocompromised mice have been used as model systems to test a number of OV, including the oncolytic herpes virus (reviewed here [65]), and OV combination therapies such as with chemotherapy or radiotherapy [64, 66]. These models are obviously limited in their scope due to the absence of an intact immune system. An alternative option widely used in OV testing is the syngeneic immunocompetent mouse system. Whilst this is an optimal system to investigate immune responses and the tumours are of murine origin, the system does not support the replication of OV making it unsuitable for the investigation of human viruses in human tumours [65]. To overcome this whilst enabling the study of human tumours, ‘humanized’ mice are used whereby irradiated mice are injected with human CD34^+^ haematopoietic stem cells (HSCs) resulting in successful engraftment of a human immune system and enabling immune responses to PDX to be assessed [67]. Tsoneva et al.[68] used such a system to determine the interaction between the oncolytic vaccinia virus with the host immune system and the subsequent effect on tumour growth alone and in combination with anti‐CTLA4 antibody. Whilst these models can be valuable, their usefulness is still limited by the unavailability of HLA ‐matched immune and tumour cells, and the inability of OVs derived from human viruses to replicate in mouse tissues. Work is underway to mitigate against these issues by improving methods to expand HSCs from patients to allow for a matched immune and tumour environment and/or through manipulating the mouse system to reduce cross‐ reactivity between mouse and human systems [69, 70].

As alternatives to the use of mice– patient chimeric system, other derived models such as organoid‐ or patient ‐derived explants (PDEs) have been effectively used for the study of OV. PDEs have proven useful as the tumour borders can be cut precisely to include both tumour and healthy tissues, thereby allowing the demonstration that a given OV targets the tumour specifically. Upon infection, it is possible to observe viral transgene expression to prove viral replication and the tissue can be cut and stained for further analysis. Whilst PDEs have been used for OV validation in a variety of settings [71– 74], their usefulness is limited in that they are only viable for a maximum of 72 h post‐excision. Organoids present an interesting and potentially useful alternative as these recapitulate the organ from which they are derived, can be produced from both healthy and tumour tissue, thereby allowing for direct comparisons. Moreover, it may be possible to incorporate autologous immune cells into organoid systems, providing a more complete model, which recapitulates the patient and their own immune system, to test novel therapies. Whilst relatively unexplored for the study of OV to date, organoids have been infected with different viruses to assess pathogenicity [ 75]. Zhu et al.[76] used organoids to demonstrate the ability of the Zika virus (ZIKV) to selectively replicate in glioblastoma stem cells (GSC), but not differentiated glioblastoma cells resulting in cell death of the GSC leading to loss of self‐ renewal and proliferation. Recently, pancreatic organoids have been utilized as a screening platform to determine infectivity, selectivity and sensitivity to oncolytic adenovirus infection [77]. The potential of this system to aid in the preclinical testing of future OVs is evident.

## CONCLUDING REMARKS

Considerable evidence now points towards an additive or even synergistic potential of OVs and immunotherapies, either as a combination therapy or as one ‘Trojan horse’ therapy, where the immunotherapy is encoded within the OV genome. There exists a plethora of viral platforms, targeting strategies and immunological payloads that can be combined into highly advanced complex therapies for future clinical translation. Defining suitable models to enable high‐throughput evaluation of these therapeutics and optimize combinations remains a challenge. Combinations of advanced models based on ex vivo evaluation in clinical isolates with increasingly sophisticated in vivo models will be required to define optimized and patient‐personalized therapies moving forward.

## CONFLICT OF INTEREST

The authors have no disclosures to make.

## Supporting information

Table S1
