# Supplementary material for: Pouring petrol on the flames: Using oncolytic virotherapies to enhance tumour immunogenicity
Source: Immunology. 2021 Mar 28;163(4):389–98. doi: 10.1111/imm.13323 (PMC8274202; doi:10.1111/imm.13323)
Supplement: Supplementary file 1 — Table S1 [file IMM-163-389-s001.docx]

**Supplemental Table 1: Combinations of oncolytic virotherapies (OV) and Immune Checkpoint Inhibitors undergoing clinical evaluation.**
